# Supplementary material for: miR-199a Downregulation as a Driver of the NOX4/HIF-1α/VEGF-A Pathway in Thyroid and Orbital Adipose Tissues from Graves′ Patients
Source: Int J Mol Sci. 2021 Dec 23;23(1):153. doi: 10.3390/ijms23010153 (PMC8745087; doi:10.3390/ijms23010153)
Supplement: Supplementary file 1 [file ijms-23-00153-s001.zip › ijms-1469456-supplementary.pdf]

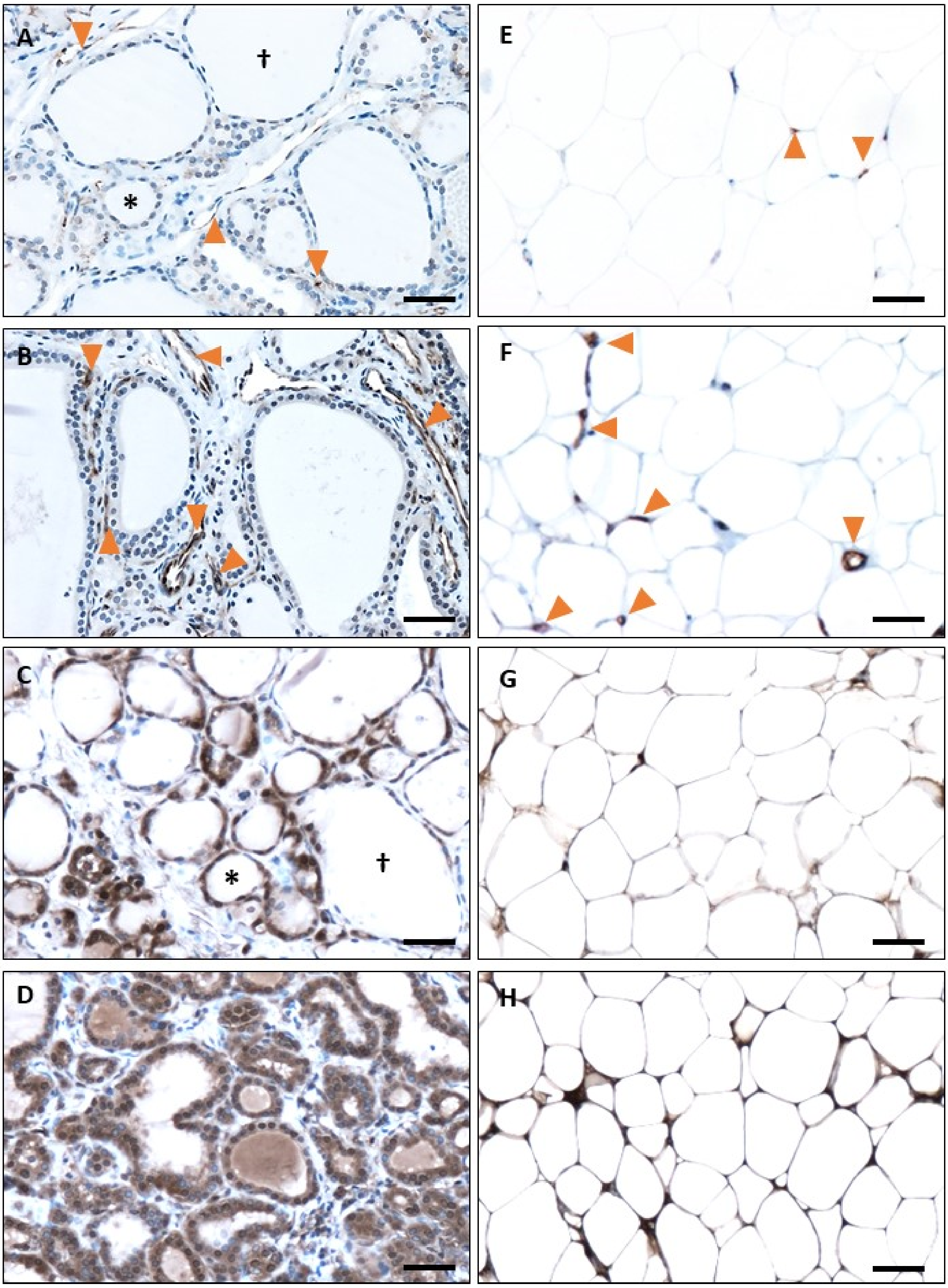

Supplemental Figure S1. CD31 and HNE immunostaining in GD/GO tissues. In a normal thyroid (A, C), the angio-follicular units (AFU) present an important heterogeneity (12;18). Active AFUs (*) have small follicular lumina lined by cylindrical cells and are richly vascularized. On the contrary, hypofunctioning AFU (†) are large with an abundant colloid and a flat epithelium and are surrounded by a hypotrophic microcirculation (48). GD thyroids (B, D) present mainly hyperactive follicles. (A-B, E-F) CD31 staining of capillaries (orange arrowheads) on 5 µm thick paraffin sections of thyroids and orbital fats. Capillaries were predominant in GD thyroid (B) and GO orbital fat (F) compared to control thyroid (A) and control orbital fat (E). (C-D, G-H) HNE staining demonstrating a higher oxidative stress in GD thyrocytes (D) and GO orbital adipocytes (H) in comparison to corresponding control tissues (C and G). *; active AFU, †; hypofunctional AFU, arrowheads; capillaries.

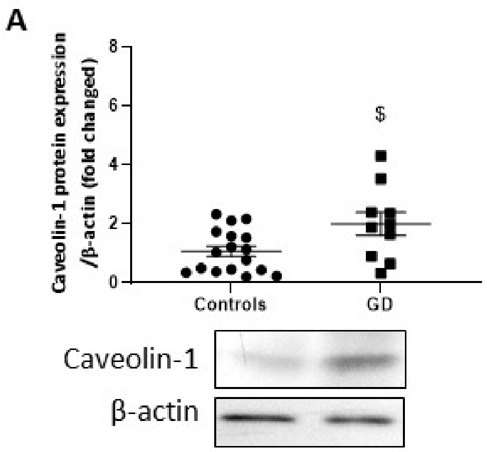

Supplemental Figure S2. **Upregulation of Caveolin-1 in GD thyroids**. Caveolin-1 protein, a main actor in signalling compartmentation and thyrocytes redox status, was significantly increased in GD thyroids versus controls. Data represent the mean +/- SEM from 17 control and 10 GD thyroids. $ $p<0.05$ compared to controls. Densitometric values were normalized against β-actin. Western blots shown are representative of both conditions.

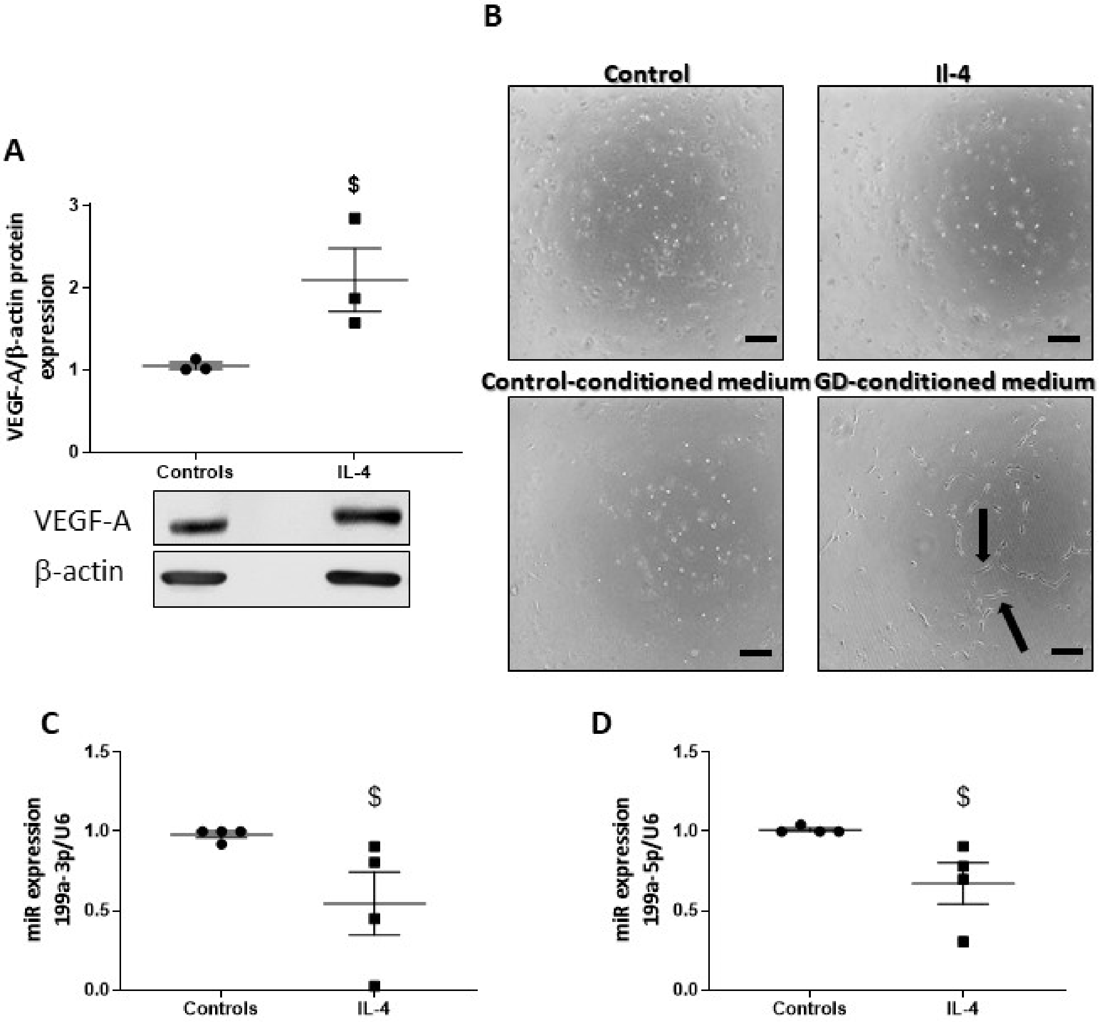

Supplemental Figure S3. In order to mimic the inflammatory context of GD, primary cultures of thyrocytes were treated with IL-4. First, human primary thyrocytes were cultured without (control) or with IL-4 to mimic GD. After 24 hours, media were collected and mixed with microvascular endothelial cells and Matrigel during 48 hours. (A) VEGF expression was significantly increased in cultured thyrocytes treated with IL-4. (B) Tert-immortalized microvascular endothelial cells (TIME), were suspended in the presence of medium from IL-4 treated thyrocytes or from untreated thyrocytes (GD-conditioned medium vs Control) or directly treated with IL-4, then mixed with Matrigel (Growth factor-free) to allow a 2D culture. After Matrigel polymerisation at 37°C during 15 minutes, microvascular endothelial cell medium was added on the top of the Matrigel/Cell mixture. Tube formation was assessed after 48 hours of culture in Matrigel. The pro-angiogenic effect, evaluated by the observation of endothelial cells alignment, appeared more pronounced in cells exposed to GD-conditioned medium. Endothelial cells, having received medium that has not been in contact with thyrocytes, in presence or absence of IL-4, did not show any tube-formation ability. However, GD-conditioned medium induced tubes formation (arrows) of endothelial cells as compared to control. (C-D) miR-199a-3p and -5p were significantly reduced in human thyrocytes treated with IL-4. The results are expressed as the mean ± SEM from at least 3 independent experiments.

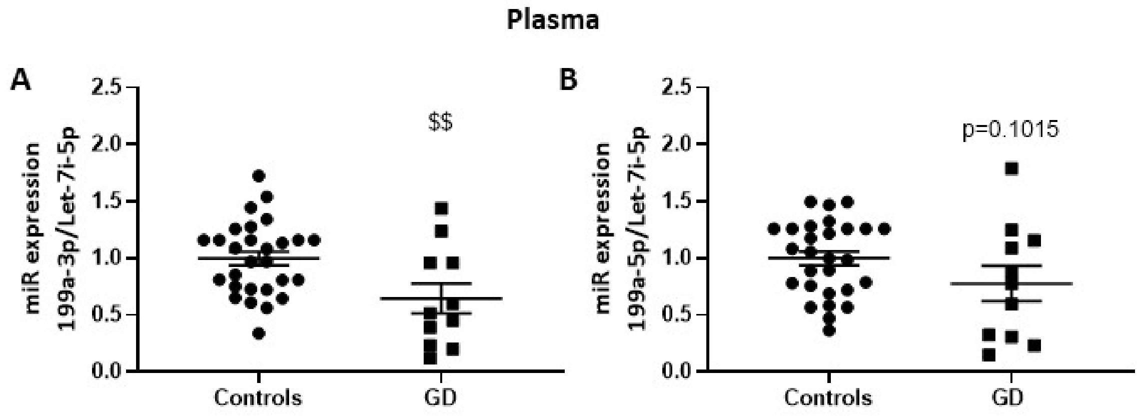

Supplemental Figure S4. Expression of miR-199a-3p and -5p in plasma of GD patients. Plasma collected from GD and control patients were analyzed. Circulating miR-119a-3p and -5p were evaluated by RT-qPCR, Let-7i-5p miR was used to normalize the data. A significant reduction of miR-199a-3p levels and a decreasing trend were observed in plasma of GD patients vs controls patients. Data represent the mean +/- SEM from 28 controls and 11 GD plasma samples. $ p<0.05 compared to controls.
